# Supplementary figures and images for: The late endosome-resident lipid bis(monoacylglycero)phosphate is a cofactor for Lassa virus fusion
Source: PLoS Pathog. 2021 Sep 7;17(9):e1009488. doi: 10.1371/journal.ppat.1009488 (PMC8448326; doi:10.1371/journal.ppat.1009488)

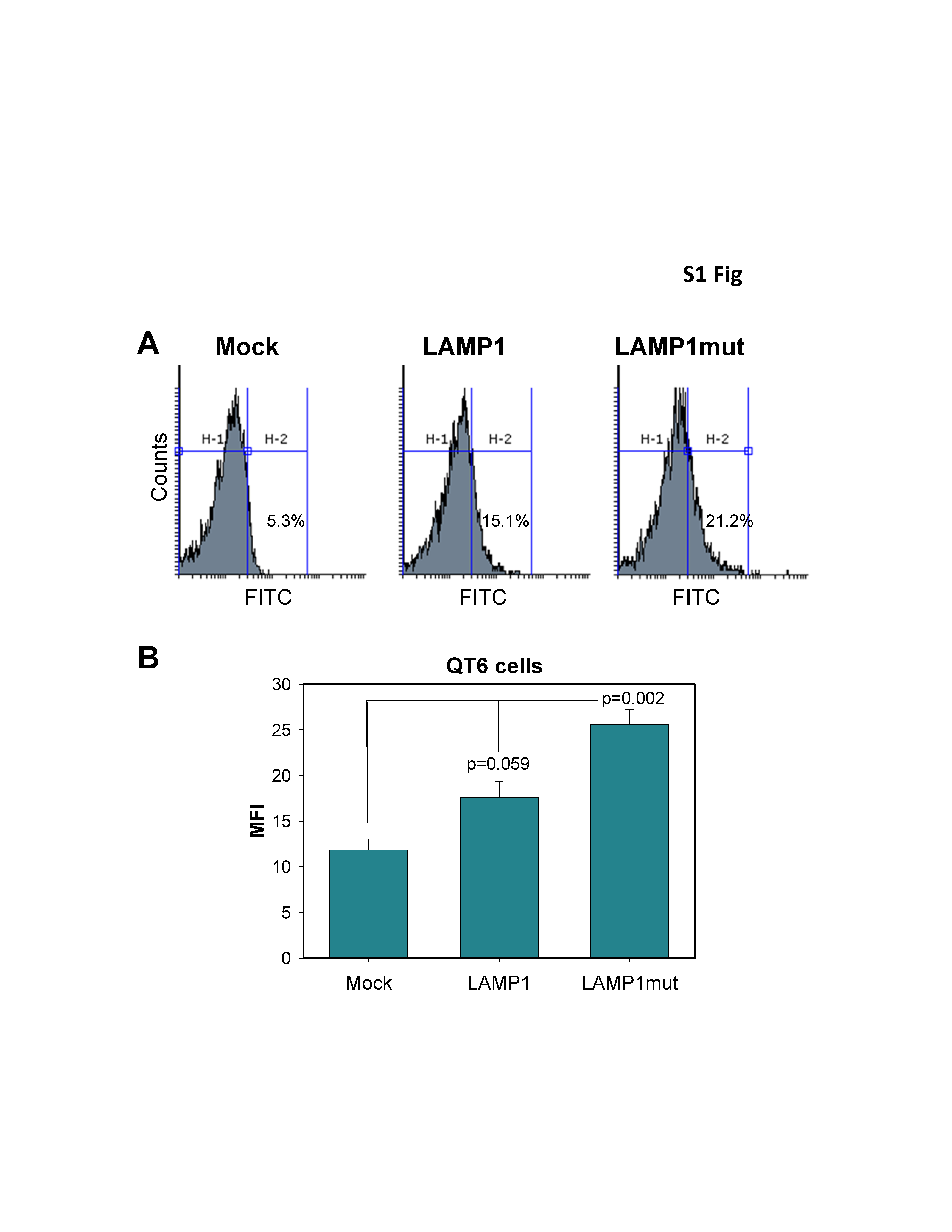

Supplement: S1 Fig — QT6 cells were mock transfected or transfected with human LAMP1 or LAMP1 mutant. Cell surface expression was measured by flow cytometry after staining with FITC-conjugated anti-LAMP1 antibody. (A) Histograms of LAMP1 signals from untransfected QT6 cells and cells expressing wild-type or mutant human LAMP1 from a representative experiment. (B) Average MFI values for cells in panel (A) from three independent experiments. Error bars are SEM. (TIF) [file ppat.1009488.s001.tif]

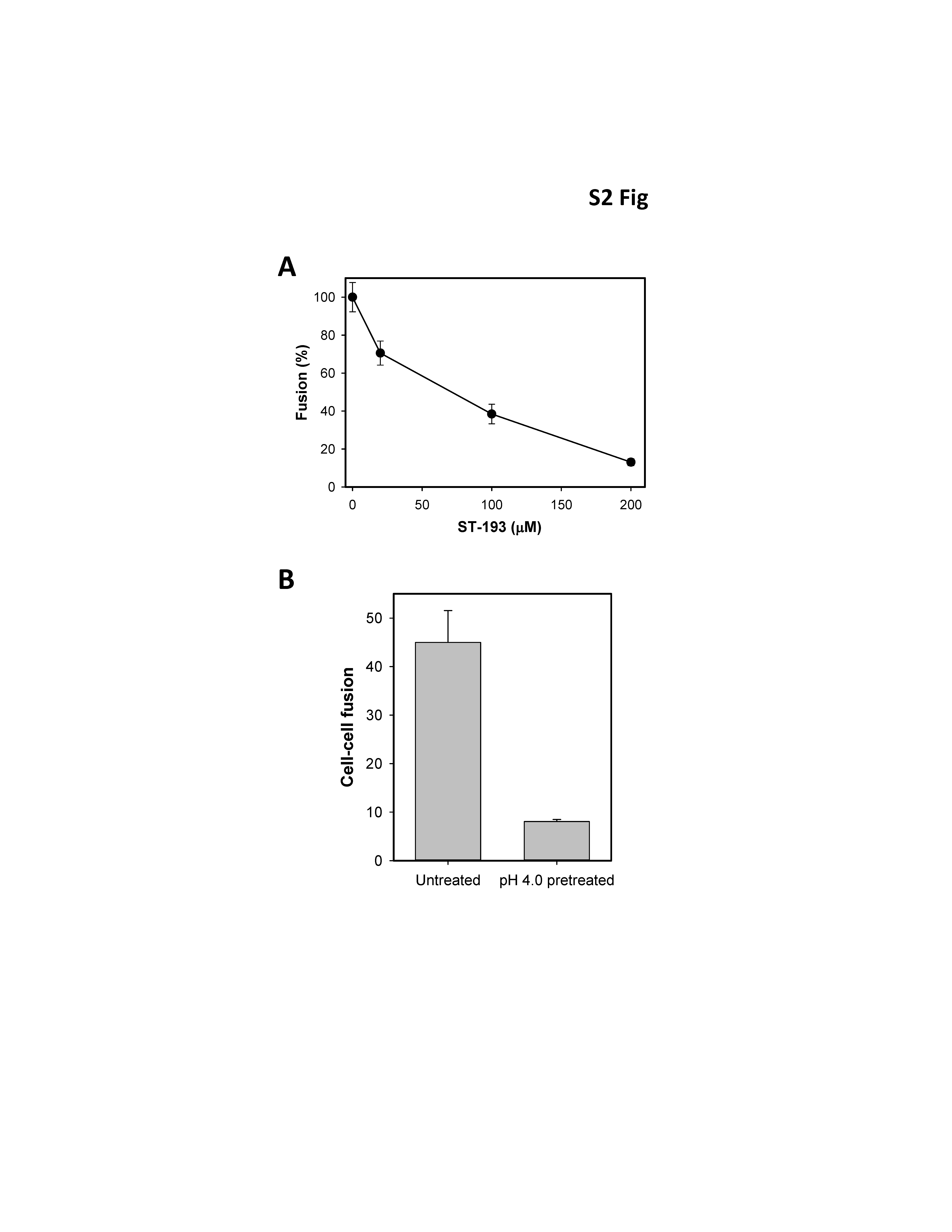

Supplement: S2 Fig — (A) Dose-dependent inhibition of LASV GPC-mediated cell-cell fusion by the arenavirus fusion inhibitor ST-193. LASV GPC-expressing COS7 cells (loaded with calcein-AM) and HEK293T cells transfected with LAMP1mut (loaded with CMAC) were mixed at a 1:1 ratio, adhered to poly-lysine coated coverslips and incubated for 30 min at room temperature. Cell fusion was triggered by exposure to pH 6.2 for 10 min at room temperature (suboptimal trigger) in the presence or absence of the indicated concentration of ST-193. The results are means and SEM from three independent experiments. (B) GPC-expressing COS7 cells were pretreated with a pH 4.0 buffer for 10 min at 37°C followed by co-incubation with target HEK293T cells for 10 min at neutral pH, room temperature, to establish cell-cell contacts. Effector-target cell complexes were then exposed to pH 6.2 for 10 min at room temperature and further incubated at neutral pH for 1 h at 37°C. The results are means and SEM from three independent experiments. (TIF) [file ppat.1009488.s002.tif]

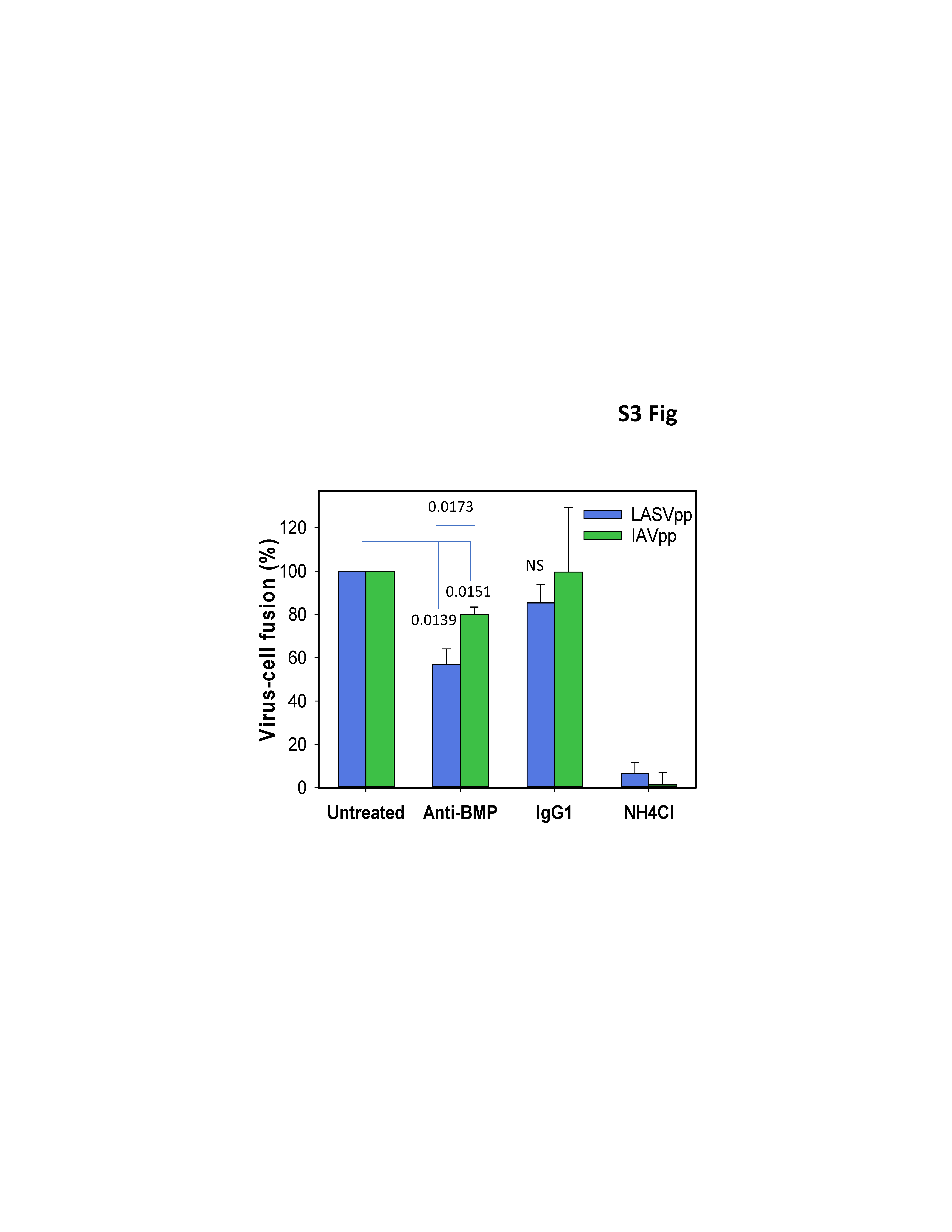

Supplement: S3 Fig — Human A549 cells were starved for 6 h and incubated with 50 μg/ml of anti-BMP or control IgG1κ antibodies for 15 h to allow antibody internalization by fluid-phase uptake before infecting with LASVpp or IAVpp. Virus-cell fusion was measured using a BlaM assay. Control samples were treated with 70 mM NH4Cl to block endosomal entry of viruses. The results are means and SD from 2 independent experiments performed in duplicates. (TIF) [file ppat.1009488.s003.tif]

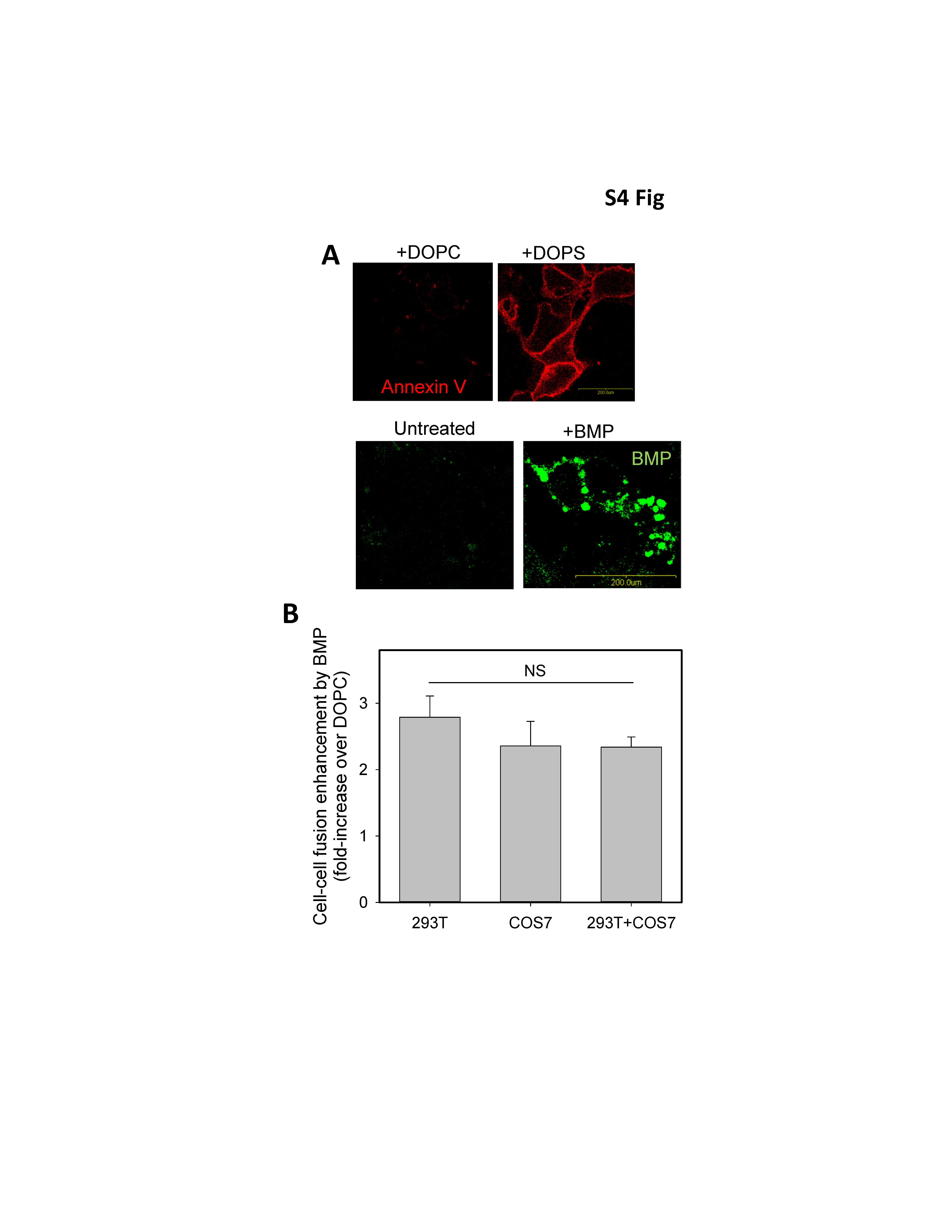

Supplement: S4 Fig — (A) Top: Representative images of Annexin V-stained COS7 cells. Cells were pretreated with 10 μg/ml DOPC (negative control) or DOPS in BSA for 20 min at room temperature, washed and stained with 5 μg/ml of AlexaFluor647-labeled Annexin V (ThermoFisher) for 1 h at 4°C. Bottom: Cells were pretreated with 10 μg/ml BMP in BSA or mock treated, washed, fixed with 4% PFA and incubated with anti-BMP antibody (1:250 dilution) for 1h at 4°C, followed by incubation with goat anti mouse AlexaFluor488-conjugated second antibody (1:1000 dilution). Images were acquired on a Fluoview300 microscope (Olympus, Melville, NY), using an UPlanApo 60X/1.20NA water-immersion objective and standard eGFP and Cy5 filter cubes for Annexin V and BMP immunostaining, respectively. (B) Effect of BMP on cell fusion upon incorporation into target or effector cells. GPC-expressing COS7 cells or target HEK293T cells were pretreated in suspension with either DOPC or BMP at 10 μg/ml for 20 min at room temperature. Cells were washed mixed with target or effector cells, respectively, and allowed to adhere to glass slides for 15 min at room temperature. Fusion was triggered by exposure to pH 5.0 for 20 min, 37°C. The results are plotted as fold-increase in cell-cell fusion by BMP relative to DOPC. Data are means and SEM from four independent experiments. (TIF) [file ppat.1009488.s004.tif]

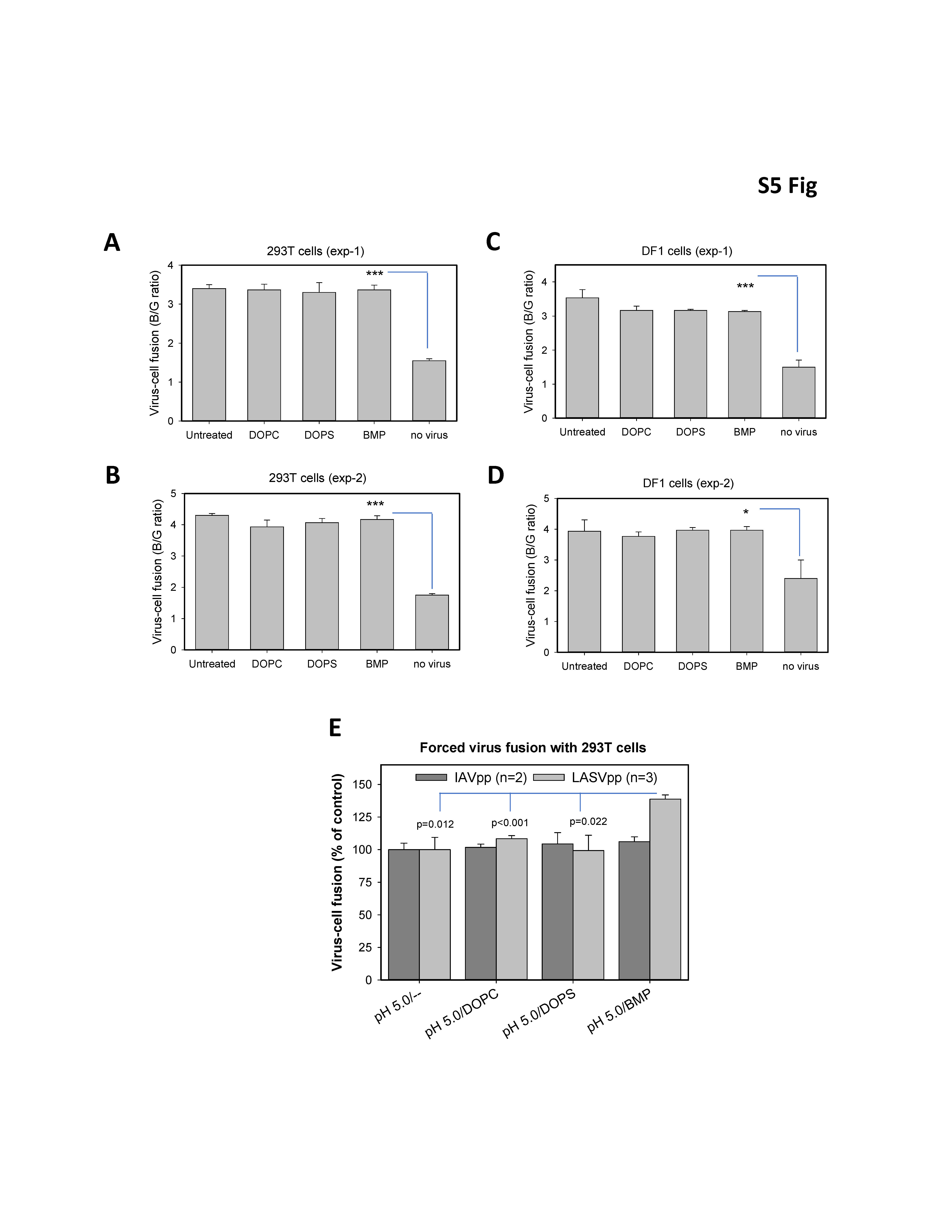

Supplement: S5 Fig — (A, B) Two independent experiments showing the effect of exogenously added DOPC, DOPS and BMP (40 μg/ml in BSA-containing PBS) on LASVpp fusion with HEK293T cells. The LASVpp fusion under these conditions was measured by a BlaM assay using the blue/green ratio of fluorescence of a BlaM substrate that was loaded into cells. (C, D) Same as in panel A, but showing the result of two experiments of LASVpp fusion with avian DF-1 cells. No significant changes in the BlaM signal were observed after cell pretreatment with any of the lipids. (E) Lipid-dependence of forced pseudovirus fusion with the plasma membrane. HEK293T cells were pretreated with 0.2 μM BafA1 for 1h at 37°C, washed and spinoculated with LASVpp or IAVpp for 30 min at room temperature. Unbound virus was removed, and cells were treated with 40 μg/ml of the indicated lipids dissolved in BSA for 10 min at room temperature. Virus fusion was then induced by exposure to pH 5.0 for 30 min at 37°C in the presence of lipids. The cells were next loaded with the BlaM substrate, and the extent of virus-cell fusion was measured by a BlaM assay after overnight incubation, as described in Materials and Methods. The results are means and SEM from 3 independent experiments (LASVpp) and two independent experiments (IAVpp). (TIF) [file ppat.1009488.s005.tif]
